# 664. Increase in the Occurrence of Carbapenem-Resistant Enterobacterales (CRE) in United States (US) Hospitals from 2019 to 2021 and Activity of Novel b-Lactam/b-Lactamase Inhibitor (BL/BLI) Combinations Against These Isolates

**DOI:** 10.1093/ofid/ofac492.716

**Published:** 2022-12-15

**Authors:** Mariana Castanheira, Timothy Doyle, Valerie Kantro, Rodrigo E Mendes, Helio S Sader

**Affiliations:** JMI Laboratories, North Liberty, Iowa; JMI Laboratories, North Liberty, Iowa; JMI Laboratories, North Liberty, Iowa; JMI Laboratories, North Liberty, Iowa; JMI Laboratories, North Liberty, Iowa

## Abstract

**Background:**

CRE isolates are considered a threat to human health. Until recently, the treatment options for CRE were limited. New BL/BLIs, such as ceftazidime-avibactam (CAZ-AVI), meropenem-vaborbactam (MVB), and imipenem-relebactam (IMI-REL), are active against most of these isolates, but these agents display some variation in activity. In this study, we evaluated the activity of these new BL/BLIs and comparator agents against CRE isolates collected in US hospitals.

**Methods:**

A total of 27,834 Enterobacterales isolates were collected in 74 US hospitals and susceptibility tested by reference broth microdilution methods. CLSI breakpoints were applied. CRE isolates resistant to imipenem or meropenem were submitted to whole genome sequencing and data analysis for the detection of β-lactamases.

**Results:**

CRE comprised 0.9% (261) of the isolates. An increase in CRE prevalence was noted in 2021 (1.1%) compared to 2019 (0.8%) and 2020 (0.9%; *p*=0.06). This increase was noted in isolates from bloodstream infections and among patients hospitalized with pneumonia; a slight decrease was observed among urinary tract infections. Among the CREs, an increase in *E. cloacae*, *K. aerogenes*, and less common Enterobacterales species was observed. CRE isolates included 8 species in 2019/2020 and 12 species in 2021. Most CRE isolates produced KPC enzymes, mainly KPC-2 and KPC-3, but a decline was noted over time, from 73.8% in 2019 to 67. 5% in 2020 and 54.1% in 2021. Conversely, MBLs increased from 3.8% in 2019 to 18.4% in 2021. MBLs were mainly NDM-1/-5, but also IMP-27 and IMP-4. OXA-48-like enzymes without NDM were detected among 8 isolates. Overall, CAZ-AVI inhibited 86.6% of CRE isolates. When comparing the activity of 3 new BL/BLIs tested against 2021 isolates, CAZ-AVI was active against 78.6% of the CRE isolates whereas MVB inhibited 76.5% and IMI-REL inhibited 73.3%.

**Conclusion:**

An increase in CREs driven by an increase in MBL-producing isolates was noted in 2021. This increase is worrisome since there are no effective therapeutic options against MBL producers. CAZ-AVI was the most active agent against CREs, but the increase in MBLs should be closely monitored. New therapies against these isolates are urgently needed.

**Disclosures:**

**Mariana Castanheira, PhD**, AbbVie: Grant/Research Support|Cidara: Grant/Research Support|GSK: Grant/Research Support|Melinta: Grant/Research Support|Pfizer: Grant/Research Support|Shionogi: Grant/Research Support **Timothy Doyle, MS**, AbbVie: Grant/Research Support **Valerie Kantro, BA**, AbbVie: Grant/Research Support|GSK: Grant/Research Support|Melinta: Grant/Research Support|Shionogi: Grant/Research Support **Rodrigo E. Mendes, PhD**, AbbVie: Grant/Research Support|Cidara: Grant/Research Support|GSK: Grant/Research Support|Melinta: Grant/Research Support|Nabriva Therapeutics: Grant/Research Support|Office for Assistant Secretary of Defense for Health Affairs: Grant/Research Support|Pfizer: Grant/Research Support|Shionogi: Grant/Research Support|Spero Therapeutics: Grant/Research Support **Helio S. Sader, MD, PhD**, AbbVie: Grant/Research Support|Cidara: Grant/Research Support|Melinta: Grant/Research Support|Nabriva Therapeutics: Grant/Research Support|Pfizer: Grant/Research Support.

